# Antiplatelet Therapy Post Coronary Artery Bypass Grafting: A survey of practice at UK Cardiothoracic Units

**DOI:** 10.1186/1749-8090-10-S1-A299

**Published:** 2015-12-16

**Authors:** Yama Shoaib Haqzad, Alexandra Woods, Mubarak Chaudhry, Mahmoud Loubani

**Affiliations:** 1 Department of Cardiothoracic Surgery, Castle Hill Hospital, Cottingham, HU16 5JQ, UK

## Background/Introduction

Bleeding complications and perioperative cardiovascular events are strongly influenced by the management of antithrombotic therapy before and after Coronary Artery Bypass Grafting (CABG). Early thrombosis is a major cause of vein graft attrition after CABG1 with occlusion rates ranging between 5 - 26% 2. There is scarce evidence in dual antiplatelet therapy post elective CABG.

## Aims/Objectives

The primary objective of this survey was to establish the variations in antiplatelet therapy post CABG in all cardiothoracic units across the United Kingdom (UK).

## Method

A comprehensive list of all cardiothoracic units across the UK was obtained from the Society for Cardiothoracic Surgery (SCTS). 41 units were identified in England, Scotland, Wales and Northern Ireland. Questionnaire regarding antiplatelet therapy was developed and administered to the on call cardiothoracic registrars over a 3 day period. Data was analysed using Microsoft Excel.

## Results

All 41 centres responded to the questionnaire. 300 mg Aspirin (ASA) was given Per Rectum 6 hours post CABG in 66% of the units while surgeon specific variation existed in 24% of the units and it was not given in 10% of the units. Post elective CABG, 51% of the units gave isolated ASA 75 mg for life while variation between consultants within the unit existed in 27%, dual antiplatelet therapy was used in 10% and varying doses of ASA administered in 7%. In post CABG patients with previous Coronary Stents or recent Acute Coronary Syndromes 51% of the units added 75 mg of Clopidogrel, 5% added ticagrelor (180 mg loading then 90 mg twice daily) to ASA while 2% only gave Aspirin with variable practice between surgeons within the same unit in 37% of the units. Variation in practice existed in 85% of the units in this survey.

## Discussion/Conclusion

This survey highlights a diverse practice in antiplatelet therapy between cardiothoracic units across the UK. There is also variation in practice between the consultants within each unit. Comprehensive survey of individual cardiothoracic surgeons across the UK may highlight.

